# Late-onset unexplained seizures are associated with cognitive impairment and lower amygdala volumes

**DOI:** 10.1093/braincomms/fcaf050

**Published:** 2025-01-31

**Authors:** Rani A Sarkis, Janet Orozco, Hernan Nicolas Lemus, Alexis Hankerson, Lei Liu, Alice D Lam, Emily Johnson, Steven Stufflebeam, Anand Viswanathan, Rebecca E Amariglio, Mallika Purandare, Patrick Trouten, Geoffrey S Young, Joseph J Locascio, Page B Pennell, Gad A Marshall

**Affiliations:** Department of Neurology, Brigham and Women’s Hospital, Harvard Medical School, Boston, MA 02115, USA; Department of Neurology, Brigham and Women’s Hospital, Harvard Medical School, Boston, MA 02115, USA; Department of Neurology, Brigham and Women’s Hospital, Harvard Medical School, Boston, MA 02115, USA; Department of Neurology, Brigham and Women’s Hospital, Harvard Medical School, Boston, MA 02115, USA; Department of Neurology, Brigham and Women’s Hospital, Harvard Medical School, Boston, MA 02115, USA; Department of Neurology, Massachusetts General Hospital, Harvard Medical School, Boston, MA 02114, USA; Department of Neurology, Johns Hopkins University School of Medicine, Baltimore, MA 21205, USA; Department of Radiology, Massachusetts General Hospital, Harvard Medical School, Boston, MA 02114, USA; Harvard-MIT Health Science and Technology, Massachusetts Institute of Technology, Boston, MA 02139, USA; Department of Neurology, Massachusetts General Hospital, Harvard Medical School, Boston, MA 02114, USA; Department of Neurology, Brigham and Women’s Hospital, Harvard Medical School, Boston, MA 02115, USA; Department of Neurology, Massachusetts General Hospital, Harvard Medical School, Boston, MA 02114, USA; Department of Neurology, Brigham and Women’s Hospital, Harvard Medical School, Boston, MA 02115, USA; Department of Neurology, Brigham and Women’s Hospital, Harvard Medical School, Boston, MA 02115, USA; Department of Radiology, Brigham and Women’s Hospital, Harvard Medical School, Boston, MA 02115, USA; Department of Neurology, Massachusetts General Hospital, Harvard Medical School, Boston, MA 02114, USA; Department of Neurology, University of Pittsburgh School of Medicine, Pittsburgh, PA 15261, USA; Department of Neurology, Brigham and Women’s Hospital, Harvard Medical School, Boston, MA 02115, USA; Department of Neurology, Massachusetts General Hospital, Harvard Medical School, Boston, MA 02114, USA

**Keywords:** geriatric, elderly, epilepsy, nonlesional, epilepsy

## Abstract

Late-onset epilepsy has been linked with accelerated cognitive decline and a higher risk of dementia. In this study, we sought to characterize the cognitive profile of participants with late-onset unexplained epilepsy and compare their MRI findings to healthy controls, to better understand underlying disease mechanisms. We recruited participants with at least one new-onset unexplained seizure at age 55 or later, without cortical lesions on MRI, within 5 years of the first seizure. We administered a neuropsychological battery to generate Preclinical Alzheimer Cognitive Composite and composite scores for delayed verbal recall, processing speed and executive function. We held a consensus meeting to determine whether the participants fulfilled criteria for mild cognitive impairment. An MRI volumetric analysis of hippocampal, amygdalae, and white matter hyperintensity volume was performed and compared to 353 healthy controls from the Harvard Aging Brain Study. On late-onset unexplained epilepsy participants, we also obtained 24-h EEG recording. Seventy participants were recruited, mean age 71.0 ± 7.0 years, 49% female, 15.6 ± 3.0 years of education. Impaired cognition (*z*-score ≤ −1.5) for late-onset unexplained epilepsy included the following: 15.9% for Preclinical Alzheimer Cognitive Composite -5, 23.2% for delayed verbal recall, 15.6% for processing speed and 7.5% for executive function. Seventeen percent were found to have mild cognitive impairment. Late-onset unexplained epilepsy participants who were drug resistant were more likely to have cognitive impairment (50% vs. 9%). When controlling for age, sex and race, late-onset unexplained epilepsy group had lower left AV (%; β = −0.003, *P* = 0.0016), right AV (%) (β = −0.003, *P* = 0.01), and log-transformed WMV (mm^3^; β = −0.21, *P* = 0.03) compared with Harvard Aging Brain Study (HABS); there were no differences in left or right HV between groups. EEG captured epileptiform abnormalities in 49% late-onset unexplained epilepsy participants, with a left temporal predominance (54%). In this single-site study of prospectively enrolled participants with late-onset unexplained epilepsy, we show that individuals with late-onset unexplained epilepsy exhibit cognitive impairments, mostly in verbal memory, and temporal dysfunction with left-sided predominance. Neuroimaging, when compared with healthy controls, shows lower amygdalae and white matter hyperintensity but not hippocampal volumes suggesting that the amygdalae is one of the earliest sites involved in the disease. The results also highlight the importance of seizure control given the association between mild cognitive impairment and drug-resistant epilepsy. Future studies extending these findings to Alzheimer’s disease biomarkers and longitudinal follow-up will inform predictors of cognitive decline.

## Teaser

Sarkis *et al*. prospectively recruited adults with late-onset unexplained seizures and found a prevalence of mild cognitive impairment of 17% and cognitive impairments mostly in verbal memory. Epileptiform abnormalities were mostly left temporal, while volumetric analysis showed lower amygdalae volumes but no difference in hippocampal volumes compared with controls.

## Graphical Abstract

**graphical abstract:**
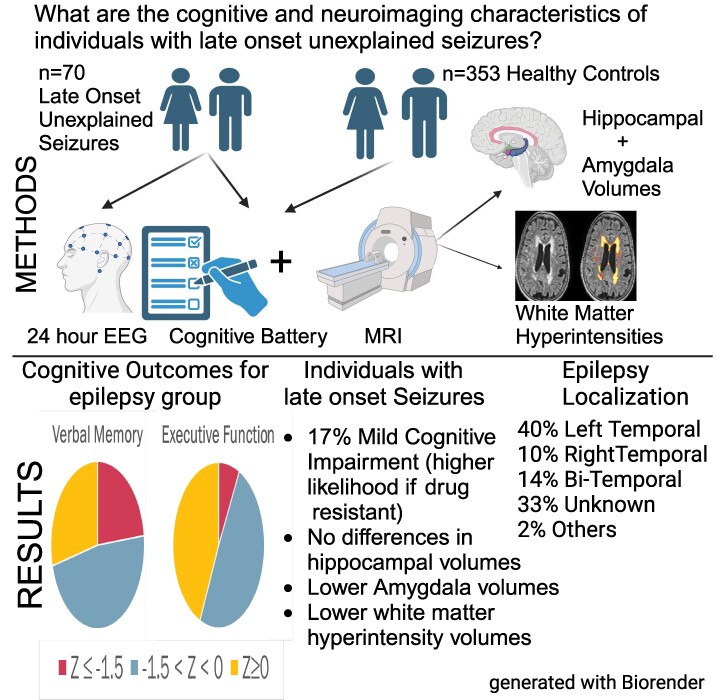

## Introduction

The incidence of epilepsy peaks later in life starting at the age of 50,^1^ and the prevalence of epilepsy by the age of 75 is twice the rate of younger adults.^2^ As the global population ages, the prevalence of late-onset epilepsy will increase, as will its medical and financial burden on patients, their caregivers, and the healthcare system.

The most common aetiologies for late-onset epilepsy are cerebrovascular and neoplastic; however, 33–53% of individuals with late-onset epilepsy do not have a clear cause^3,4^ and are considered to have ‘late-onset unexplained epilepsy (LOUE)’. The predominant hypothesis is that most cases of LOUE are due to underlying occult cerebrovascular disease,^5^ while a subpopulation may be secondary to neurodegenerative pathologies such as amyloid-β (as seen in preclinical Alzheimer’s disease).^6^ In this context, some experts consider LOUE to be an epileptic variant of Alzheimer’s disease.^7^ Epidemiologic studies have highlighted the negative outcomes for late-onset epilepsy with a 3-fold increased risk of stroke,^8^ a risk of accelerated cognitive decline^9^ and a 2- to 3-fold increased risk of dementia^10,11^; however, these studies included participants with various aetiologies. Studies examining cognition in participants with LOUE have been limited to date, with evidence of worse memory and executive function scores when compared with age-matched controls,^12^ and a decline in verbal memory and the mini mental status exam at 1 year.^13^

Retrospective analyses from our team and others indicate that the temporal lobe is the predominant location of epileptiform abnormalities on EEG in LOUE.^14,15^ Complimenting this, FDG-PET findings also highlight temporal lobe hypometabolism in LOUE.^16^ This is noteworthy since mesial temporal structures such as the amygdalae and hippocampus are some of the early sites affected by buildup of the neurodegenerative proteins amyloid-β^17^ and phosphorylated tau,^18^ which have also been found to be pro-epileptogenic.^19^

In the current prospective study, we sought to examine the cognitive profile of participants with LOUE and evaluate their cerebrovascular risk factors. We explored evidence for early neurodegenerative disease in the mesial temporal lobes by assessing hippocampal and amygdalae volumes. We hypothesized that participants with LOUE would have impaired memory scores, a higher burden of white matter hyperintensities and lower amygdalae and hippocampal volumes when compared with controls.

## Materials and methods

### Study participants

We prospectively recruited participants with late-onset unexplained seizures from epilepsy and neurology clinics at Brigham and Women’s and affiliated hospitals including the main campus and community hospitals (Faulkner Hospital, South Shore Hospital). The study was approved by the Institutional Review board at Brigham and Women’s Hospital (BWH, no. 2019P00143). Written informed consent was obtained from all participants before any study procedures were carried out according to the Declaration of Helsinki.

Inclusion criteria included at least 1 unexplained seizure with age of onset older than 55, onset of seizures within the past 5 years and a prior MRI of the brain with contrast with no identifiable cortical lesion such as cortical stroke/tumor/encephalomalacia/cavernoma (microhaemorrhages and hippocampal sclerosis were included). Participants with a remote history of seizures, with a non-lesional MRI and resolution of epilepsy (10 years seizure free and 5 years off anti-seizure medications)^20^ were eligible for enrolment if seizures recurred after the age of 55. There were no predetermined cognitive cut-offs for the LOUE group at study entry (though diagnosed dementia was excluded). None of the participants were analyzed in our prior retrospective study.^14^

Exclusion criteria included the following: non-English speaking, provoked seizures, prior diagnosis of a neurodegenerative condition, cortical stroke, central nervous system tumors, a diagnosis of dementia, suspected autoimmune encephalitis, co-morbid psychotic symptoms, seizures due to traumatic brain injury and ongoing alcohol or polysubstance abuse.

The comparison group consisted of all 353 participants older than 55 from the HABS,^21^ an ongoing study of healthy older adults with no neurologic disease recruited from the community, who underwent a similar study protocol and were within the same age range.

Data extracted included age, race, sex, handedness and years of education. All participants underwent a baseline cognitive battery consisting of the American version of the National Adult Reading Test, mini mental status exam, logical memory—delayed recall (LogMemDR) task from the Weschler memory scale,^22^ Free and Cued Selective Reminding Task,^23^ Trailmaking tests A and B (TMT A, B), category fluency (animals, fruits, vegetables), letter fluency (FAS), Digit Symbol Substitution Test^24^ and Letter Number Sequencing.^25^ Performance on each test was transformed into a *Z* score, with the HABS group used as a reference. Composite scores were then extracted for memory (average *Z* scores of LogMemDR + free and cued selective reminding task), executive functions (average *Z* scores of FAS + TMTB), processing speed (average *Z* scores of Digit Symbol Substitution Test + TMTA) and the preclinical Alzheimer cognitive composite (PACC-5),^26^ a measure optimized to detect amyloid-β related cognitive decline.^27^ The PACC-5 consists of the average *Z* score of the mini mental status exam, Digit Symbol Substitution Test, LogMemDR, category fluency, free and cued selective reminding task total score.

Participants also completed the Clinical Dementia Rating scale (CDR),^28^ which is a measure of global functioning, and the Geriatric Depression Scale (GDS)^29^ to screen for depressive symptoms. The GDS consists of 30 items with yes/no responses; scores range from 0 to 30, with higher scores indicating more severe depression. Composite scores were then categorized as normal if z-score ≥0, impaired if −1.5 < z-score <0, and mild cognitive impairment if z-score ≤−1.5. A consensus meeting which included a neuropsychologist, behavioural neurologist and epileptologist was held to determine whether the participants fulfilled criteria for mild cognitive impairment.

At study entry, the HABS participants had a global CDR of 0, GDS ≤11, mini mental status exam ≥ 27 and performed within education adjusted norms on Logical Memory.

Vascular risk factors including a history of hypertension, diabetes, smoking status and body mass index were also recorded. Apolipoprotein E (ApoE) ε4 carrier status for the epilepsy and HABS participants was determined by the presence of at least 1 ε4 allele.

### Neuroimaging

All LOUE participants also underwent a 3T brain MRI on a Siemens Prisma scanner with magnetization-prepared rapid gradient-echo, repetition time, echo time and inversion time, respectively, 2300, 2.95 and 900 milliseconds; and 1.1 × 1.1 × 1.2-mm resolution and 3D fluid attenuated inversion recovery sequences, repetition time, echo time and inversion time, respectively, 6000, 454 and 2100 milliseconds; and 1.0 × 1.0 × 1.5-mm resolution. Volumetric measurements were obtained using FreeSurfer version 5.1 (Laboratory for Computational Neuroimaging at the Athinoula A. Martinos Center for Biomedical Imaging).^30^ Three participants had cortical microhaemorrhages on MRI prior to study enrolment, and 1 participant had evidence of left mesial temporal sclerosis.

The volumetric areas of interest included the cortex volume, bilateral hippocampi and amygdalae. Each volume was divided by the total intracranial volume of each participant to adjust for head size to obtain a normalized volume. White matter hyperintensities were also quantified using a lesion prediction algorithm^31,32^ and were log-transformed to account for a positive skew.

### EEG recordings and epilepsy variables

All epilepsy participants underwent prolonged EEG monitoring. Scalp EEG electrodes were placed using the International 10–20 system with additional anterior temporal electrodes (T1, T2). Twenty-four-hour ambulatory EEG recordings were acquired with XLTEK TREX hardware (Natus Medical Inc, Pleasanton, CA, USA), sampling at 200 Hz or with Arc Apollo EEG hardware (Cadwell, Kennewick, WA, USA), sampling at 250 Hz. If participants had an epilepsy monitoring unit admission within 6 months of enrolment, then that data were used instead of the ambulatory EEG, and the first 24 h was used for analysis.

EEGs were visually reviewed separately by 2 certified epileptologists (H.N.L., R.A.S.) who marked whether the study was normal and whether it had any interictal epileptiform abnormalities, including spike waves, sharp waves and temporal intermittent rhythmic delta activity. A consensus by the 2 reviewers was reached in case of disagreement on initial review.

Epilepsy variables also included: number of anti-seizure medications, seizure semiology and whether the participant was drug resistant^33^ if they had been treated for at least 1 year. Epilepsy localization was based on seizure semiology and review of prior and current EEG findings.

### Statistics

Univariate analyses were performed to compare the demographic, clinical and neuroimaging variables between the two groups. Continuous variables were assessed for normality using the Shapiro–Wilk test. Student’s *t*-test or the χ^2^ test was used to compare the LOUE cohort to HABS for continuous and categorical variables, respectively, and the Wilcoxon rank sum test was used for non-normally distributed variables. The primary hypothesis was that the LOUE group would have lower volumes of the hippocampi and amygdalae and higher volumes of white matter hyperintensities than healthy controls.

Separate linear regression analyses were then performed with right and left hippocampal volumes (percentage of total intracranial volume), right and left amygdalae volumes (percentage of total intracranial volume) and log-transformed white matter hyperintensity volumes (mm^3^) as the dependent variables. The primary predictor of interest was group (LOUE versus HABS). Age, race and sex were used as covariates. In separate sensitivity analyses, we added (i) vascular risk factors as a covariate (smoking status, diabetes, hypertension, body mass index), and separately (ii) the mini mental status score, and the geriatric depression score and (iii) the clinical dementia rating scale.

In secondary analyses, we compared clinical and neuroimaging variables between the LOUE participants with and without mild cognitive impairment. A *P*-value <0.05 was considered statistically significant. Statistical analysis was performed using JMP version 16.2 (SAS Institute Inc., Cary, NC, USA).

## Results

### Clinical and cognitive outcomes

**Figure 1:**
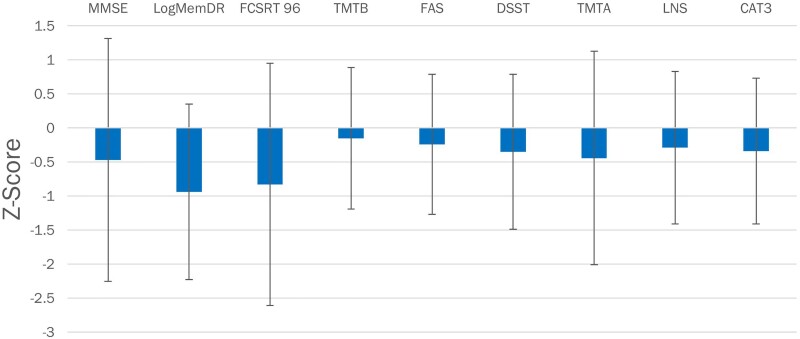
**Individual cognitive test performance in late-onset unexplained epilepsy.** *Z* transformed results on individual cognitive tests for the late-onset unexplained epilepsy participants. CAT3, category fluency 3 (*n* = 68); DSST, digit symbol substitution test (*n* = 64); FAS, phonemic fluency letters F-A-S (*n* = 68), free and cued selective reminding task 96: free and cued selective reminding test (*n* = 69); LogMemDR, logical memory delayed recall (*n* = 69); LNS, letter number sequencing (*n* = 66); MMSE, mini mental status exam (*n* = 69); TMTA, Trail Making test A (*n* = 69); TMT B, Trail making test B (*n* = 68).

**Figure 2:**
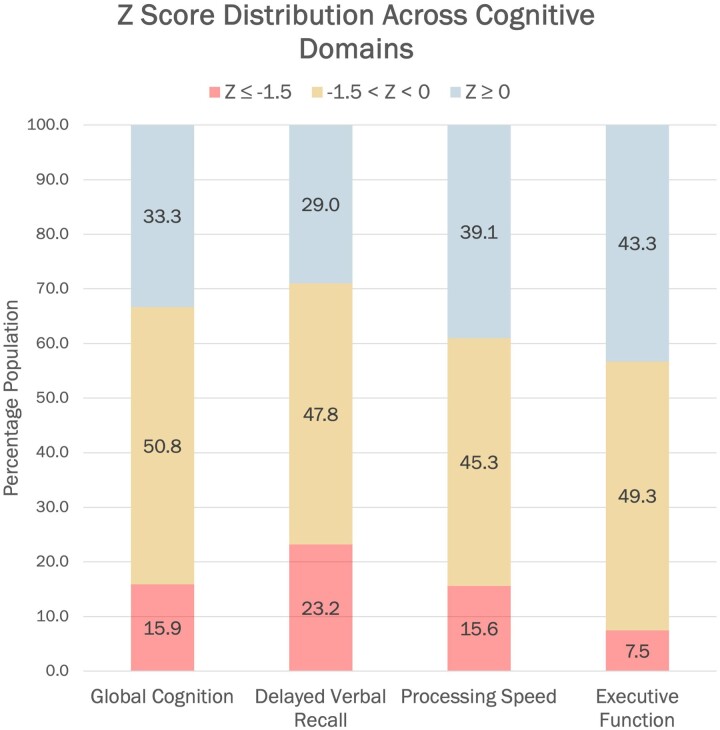
**Cognitive composite scores in late-onset unexplained epilepsy.** *Z* score distribution of the cognitive composites for the late-onset unexplained epilepsy group for Global cognition (*n* = 63), delayed verbal recall (*n* = 69), processing speed (*n* = 64) and executive function (*n* = 67).

A total of 108 LOUE participants were prospectively approached about the study between 2019 and 2024, and 70 were enrolled (Supplementary Fig. 1). Table 1 summarizes their clinical characteristics. Based on the consensus meeting, 17% were deemed to have mild cognitive impairment. The LOUE group was predominantly White (99%) and had higher depression scores on the GDS compared with the HABS control group. Rates of hypertension, diabetes and smoking were similar between the groups. In terms of handedness, 95% of the LOUE cohort was right-handed, 3% left-handed and 1% ambidextrous. The most impaired cognitive domains for the LOUE participants were those related to verbal memory (Figs 1 and 2). The global CDR scores ranged from CDR 0 (*n* = 47, 69%), to CDR 0.5 (*n* = 21, 31%), and CDR sum of boxes mean was 0.5 (range 0–4.5). LOUE participants with mild cognitive impairments were on a higher number of anti-seizure medications and were more likely to be drug resistant compared with participants without mild cognitive impairment, the neuroimaging findings and clinical characteristics were otherwise similar between both groups (Table 2).

**Table 1:** Demographic, clinical and neuroimaging characteristics of the cohorts

	LOUE (*n* = 70)	HABS (*n* = 353)	*P*-value
Age (years ± SD)	71.0 ± 7.0	72.3 ± 7.5	0.19
Sex			0.09
Female	34 (49%)	210 (59%)	
Male	36 (51%)	143 (41%)	
Education (years ± SD)	15.6 ± 3.0	15.8 ± 3.0	0.55
Ethnicity^a^			
Hispanic	3 (4%)	3 (1%)	0.056
Non-Hispanic	62 (96%)	350 (99%)	
Race^a^			<0.001
White	69 (99%)	286 (81%)	
Black	0	58 (16%)	
Asian	1 (1%)	7 (2%)	
History of hypertension	39 (56%)	154/336 (46%)	0.13
History of diabetes	3 (4%)	35/332 (11%)	0.08
Current smoker	3 (4%)	17/348 (5%)	0.82
BMI ± SD	26.5 ± 4.7	26.9 ± 4.6	0.54
GDS^a^ ± SD	5.7 ± 4.5	3.2 ± 3.2	<0.0001
AMNART	121.9 ± 9.0	120.9 ± 9.2	0.40
APOE4 carrier	13/44 (30%)	95/344 (28%)	0.79
Normalized left hippocampal volume (%)	0.248 ± 0.046 (*n* = 64)	0.247 ± 0.029	0.89
Normalized right hippocampal volume (%)	0.246 ± 0.046	0.254 ± 0.029	0.07
Normalized left amygdala volume (%)^a^	0.092 ± 0.019	0.097 ± 0.015	0.011
Normalized right amygdala volume (%)^a^	0.102 ± 0.020	0.107 ± 0.014	0.012
Normalized cortex volume (%)	27.2 ± 4.0	28.2 ± 1.6	0.0013
Log transformed white matter hyperintensity volume (mm^3^)^a^	6.86 ± 1.92 (*n* = 63)	7.50 ± 1.50	0.009

AMNART, American version of the National Adult Reading Test; BMI, body mass index; GDS, Geriatric Depression Scale; HABS, Harvard aging brain study; LOUE, late-onset unexplained epilepsy.

^a^ *P* < 0.05.

**Table 2:** Comparison of demographic and neuroimaging characteristics of LOUE participants with or without mild cognitive impairment

	MCI (*n* = 12)	Non-MCI (*n* = 58)	*P* value
Age (years ± SD)	72.8 ± 7.0	70.6 ± 7.0	0.32
Sex: female	3 (25%)	31 (54%)	0.07
Education (years ± SD)	15.4 ± 3.0	15.7 ± 3.0	0.80
No. of ASMs (range)	1.58 (1–3)	1.07 (0–3)	0.003
Drug resistant^a^	4/8 (50%)	3/32 (9%)	0.01
Epilepsy duration (years ± SD)	1.5 ± 1.0	1.6 ± 1.4	0.90
Normalized left hippocampal volume (%)	0.253 ± 0.039	0.247 ± 0.047	0.65
Normalized right hippocampal volume (%)	0.250 ± 0.037	0.246 ± 0.049	0.79
Normalized left amygdala volume (%)	0.099 ± 0.022	0.090 ± 0.019	0.16
Normalized right amygdala volume (%)	0.100 ± 0.016	0.102 ± 0.021	0.73
Normalized cortex volume (%)	26.7 ± 3.6	27.3 ± 4.1	0.62
Log-transformed white matter hyperintensity volume (mm^3^)	7.18 ± 1.19	6.79 ± 2.05	0.55

ASM, anti-seizure medication; MCI, mild cognitive impairment.

^a^If followed for at least a year.

### Neuroimaging findings

**Figure 3:**
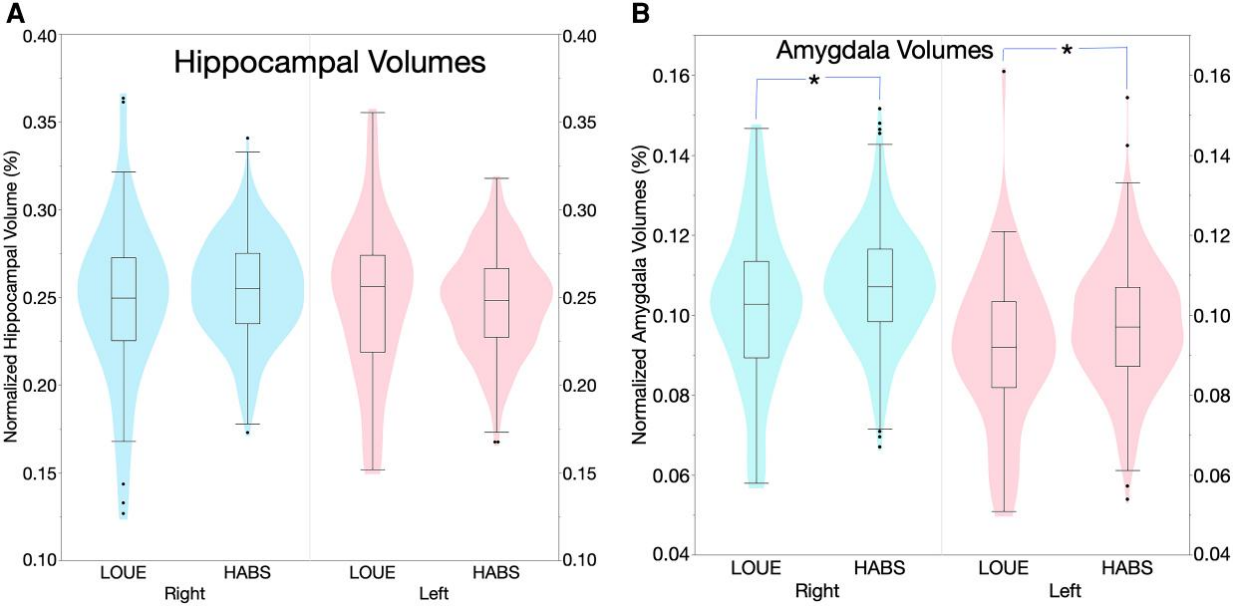
**(A, B) Amygdalae and hippocampal volumes in late-onset unexplained epilepsy versus controls.** Violin plot with embedded box plot of normalized left and right hippocampal (**A**) and amygdala volumes (%) (**B**) in LOUE (*n* = 64) versus controls from HABS (*n* = 353). *P < 0.05. Right hippocampal volume student’s *t*-test = 1.79, *P* = 0.07, left hippocampal volume Student’s *t*-test = −0.13, *P* = 0.89, right amygdala volume Student’s *t*-test = 2.25, *P* = 0.01, left amygdalae volume Student’s *t*-test = 2.56, *P* = 0.01. The plots show a wider distribution of hippocampal volumes, and on average lower left and right amygdala volumes in the epilepsy group.

**Figure 4:**
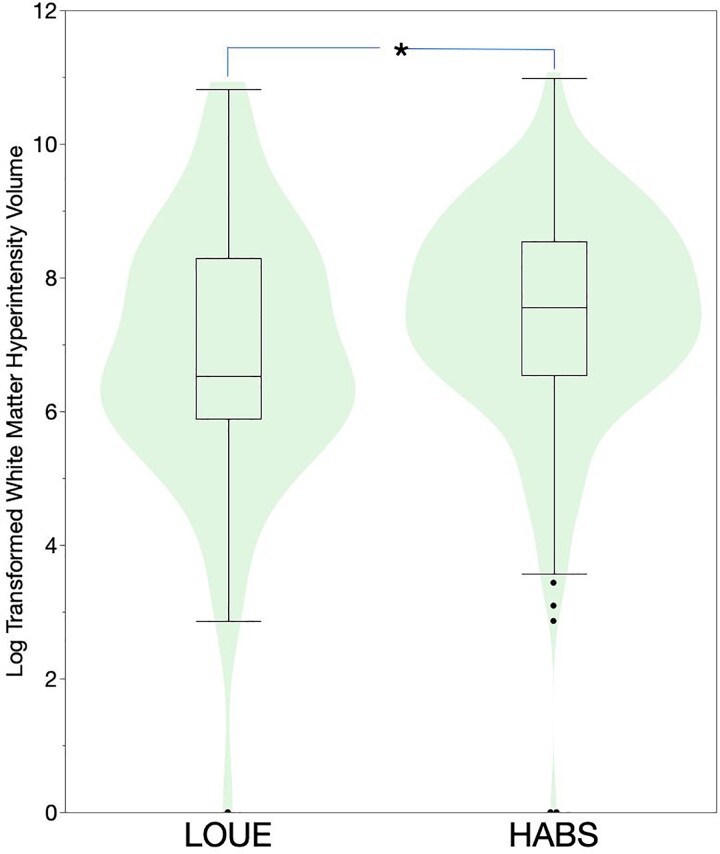
**White matter hyperintensity volumes in late-onset unexplained epilepsy versus controls.** Violin plot with embedded box plot showing the distribution of log transformed white matter hyperintensities between the LOUE (*n* = 63) and Harvard aging brain cohort (*n* = 346). *P < 0.05, Student’s *t*-test = 2.61, *P* = 0.009.

In univariate analysis, the LOUE participants were found to have no statistically significant differences in left or right normalized hippocampal volumes (%) compared with healthy controls (Fig. 3A) although there was a trend for lower right hippocampal volumes in the LOUE group. They were found to have lower right and left normalized amygdalae volumes (%; Fig. 3), and a lower volume of log-transformed white matter hyperintensities (mm^3^; Fig. 4) compared with HABS.

When controlling for age, race and sex, the univariate findings persisted: normalized left and right hippocampal volumes (%) were similar in the LOUE group compared to healthy controls [β = +0.0008, 95% CI (−0.003 to +0.005), *P* = 0.70] and [β = −0.003, 95% CI (−0.007 to +0.0008), *P* = 0.11], respectively. However, normalized left and right amygdalae volumes (%) were both lower in the LOUE group compared with controls [β = −0.003, 95% CI (−0.005 to −0.001), *P* = 0.0016], right AV (%) [β = −0.003, 95% CI (−0.005 to −0.0006), *P* = 0.01], respectively. Similarly, log-transformed white matter hyperintensity volumes were lower in the LOUE group compared with controls [LOUE group; β = −0.21, 95% CI (−0.41 to −0.02), *P* = 0.03; Supplementary material models 1–5]. The findings remained significant after controlling for vascular risk factors (Supplementary material models 6 to 10), and for controlling for the mini mental status score and the geriatric depression score (Supplementary material models 11 to 15). Finally, when controlling for the clinical dementia rating scale, the results persisted except for the right amygdala volume (*P* = 0.057; Supplementary material models 16 to 20).

### EEG and epilepsy related variables

Epilepsy characteristics and EEG findings are summarized in Table 3. Two participants had a remote history of seizures, with remission of their epilepsy and then recurrence after the age of 55.

**Table 3:** EEG and epilepsy-related characteristics of the LOUE cohort

	LOUE (*n* = 70)
Epilepsy duration (years ± SD)	1.6 ± 1.4
Seizure semiology	
Focal aware	19 (27%)
Focal with impaired awareness	33 (47%)
Focal to bilateral tonic clonic	45 (64%)
EEG	
Normal	18 (26%)
Epileptiform abnormalities	34 (49%)
Anti-seizure medications	
None	4 (6%)
Monotherapy	53 (75%)
Polytherapy	13 (19%)
Drug resistant^a^	7/40 (18%)
Epilepsy localization^b^	
Left temporal	28 (40%)
Right temporal	7 (10%)
Bitemporal	10 (14%)
Others	2 (3%)
Unknown	23 (33%)

LOUE, late-onset unexplained epilepsy.

^a^For participants treated for >1 year.

^b^Based on semiology, current and prior EEGs.

In terms of seizure semiology: 27% of LOUE participants had focal aware seizures, 47% had focal impaired awareness seizures and 64% had focal to bilateral tonic clonic seizures. The focal aware seizure semiologies included the following: sensory/motor (3), cognitive (déjà vu/depersonalization/aphasia/amnesia: 11), autonomic (flushing: 5). The anti-seizure medications used as monotherapy included the following: levetiracetam (62%), lamotrigine (21%), lacosamide (6%), brivaracetam (4%), valproic acid (6%) and gabapentin (2%).

One participant with LOUE was enrolled in the study after their epilepsy monitoring admission, while the others underwent an ambulatory EEG. In those with epileptiform abnormalities on EEG (49%), the localization was bitemporal (26%), left temporal (57%), right temporal (12%), right frontal (3%) and right frontal/right posterior quadrant (3%). One participant had a right temporal seizure captured on ambulatory EEG. Only 4 participants had an isolated seizure with no risk factors for recurrence^20^ (seizure occurred during wakefulness, no epileptiform abnormalities on EEG).

## Discussion

In this prospective study of participants with late-onset unexplained epilepsy within 5 years of disease onset, we found a 17% prevalence of mild cognitive impairment, with verbal memory being the most affected cognitive domain. EEG findings implicated the temporal lobes with left sided predominance, while neuroimaging analysis showed lower amygdalae and white matter hyperintensity volumes, but no differences in hippocampal volumes compared with a control group from the Harvard Aging Brain Study.

## Cognitive outcomes in late-onset unexplained epilepsy

Late-onset epilepsy is now a well-established risk factor for dementia, with an elevated 2- to 3-fold risk of dementia compared with persons without late-onset epilepsy. Epidemiologic studies including Atherosclerosis risk in communities (ARIC) have highlighted a course of accelerated cognitive decline in late-onset epilepsy impacting multiple cognitive domains including word fluency, verbal recall, executive function and global cognition.^34^ Cross-sectional studies have found variable results; in a study of 40 participants with LOUE, there were no differences on tests of verbal recall (Rey Auditory Verbal Learning Test), or tests of processing speed and executive function (Trailmaking test A and B) compared with controls.^35^ In a separate study of late-onset epilepsy from ARIC, using a more sensitive definition of a score <1 standard deviation below the reference to define impairment, the authors found a prevalence of memory impairment in 29.3%, executive function/processing speed impairment in 39.5% and language impairment in 39.8%.^36^ It is difficult to contrast this with the current study since the ARIC findings are confounded by the inclusion of patients with stroke and other lesions and the use of a different cut-off for impairment. The preponderance of verbal memory impairment in our study goes along with the prevalence of left temporal lobe epilepsy in the cohort. In contrast, the executive function/processing speed impairments were likely less prominent in our cohort compared with others, which may be due to the use of anti-seizure medication monotherapy (as opposed to polytherapy) and a lower burden of white matter disease.^37^

Finally, mild cognitive impairment was identified in 17% of our cohort. This LOUE subgroup could be at higher risk of accelerated decline. For comparison, the prevalence of mild cognitive impairment in community dwelling adults aged 60 to 79 years ranges between 11.5% and 15.8%, although there is significant heterogeneity between studies.^38^

In addition, this subgroup was also on a higher number of anti-seizure medications and was more likely to be drug resistant, which could in turn affect cognition. Seizure control could be a modifiable risk factor for cognitive impairment in this population. Longitudinal follow-up and biomarker data are needed to better understand the cognitive trajectory and the relationships between seizures, medication and cognition.

## Neuroimaging findings in late-onset unexplained epilepsy

In our study, we did not find differences in hippocampal volumes between the late-onset epilepsy group and controls. Our findings align with those from Gugger *et al*.^39^ who compared the MRI data of 27 late-onset epilepsy participants from the the ARIC study to controls without a history of seizures. Of note, the prevalence of stroke and dementia in this cohort was 7.4% and 14.8%, respectively. Amygdalae volumes were also not different in this study. Similarly, in a multi-site study of 23 participants with drug-resistant temporal lobe epilepsy with age of onset after 55, and average duration of epilepsy of 6.6 years, there were no differences between hippocampal volumes compared with 70 healthy controls.^40^

The hippocampus and amygdalae are of particular interest as these are early sites of accumulation of pathologic protein misfolding in neurodegenerative conditions.^17,41^ Aging-related pathologies such as phosphorylated tau and amyloid are pro-epileptogenic and could represent the aetiology of epilepsy in a subset of our cohort.^19^ To our knowledge, our study is the first to show lower amygdalae volumes in LOUE, suggesting that it might be one of the first neuroanatomical sites involved in this disease, although the magnitude of difference was small. Furthermore, we note that five participants in our study experienced autonomic auras, a semiology which can localize to the amygdalae.^42^ Our findings remained significant after controlling for relevant confounders such as global cognition, depressive symptoms^43,44^ and vascular risk factors.

Studies examining Alzheimer’s disease biomarkers in LOUE have also shown lower CSF amyloid levels^35^ suggesting higher amyloid burden in the brain and a reduction in plasma Aβ42/Aβ40 from mid to late life.^45^

In general, late-onset epilepsy tends to be a unilateral disease^14^ suggesting selective vulnerability of one temporal lobe over the other. Amyloid PET studies have shown asymmetric amyloid deposition in preclinical Alzheimer’s disease, which becomes more symmetric with cognitive decline,^46^ while asymmetric tau patterns have also been described in tau PET studies as well including a left-sided predominant subtype in 19% of patients with Alzheimer’s disease.^47^

Finally, LOUE is often thought to be due to occult cerebrovascular disease.^48^ In the ARIC study, with a diverse racial cohort, participants with late-onset epilepsy had a higher white matter hyperintensity visual rating grade, but no differences in volume when compared with controls.^49^ In our cohort, we found lower white matter hyperintensity volume in the epilepsy group compared to controls. When evaluating vascular risk factors between our groups, there was a higher prevalence of hypertension in the epilepsy group and diabetes in the control group, but these did not reach statistical significance. There were however racial differences between the groups, with the epilepsy cohort predominantly consisting of White participants. It is possible that these racial differences underly these results.^50^ It will be important for future studies to re-examine the contribution of cerebrovascular disease in cohorts that are balanced with regard to race.

## Epilepsy-related characteristics in late-onset unexplained epilepsy

EEG findings in our study highlight LOUE as a temporal disease, with most epileptiform abnormalities affecting the left temporal lobe. Our team showed similar findings in a retrospective review of LOUE patients.^14^ The left temporal involvement could indicate an underlying vulnerability with aging^51^ or a certain bias because patients are more likely to be symptomatic (e.g. presenting with aphasia) and seek medical attention. This left sided predominance was not noted in a study of Alzheimer’s disease patients using magneto-encephalography,^52^ and it was also not present in the neuroimaging data with both amygdalae having lower volumes compared with controls and no differences in hippocampal volumes between the groups. Another possibility is that left temporal epileptiform discharges are more likely to be picked up on EEG.

LOUE is a medically responsive entity. In our study, 82% of participants were controlled with medications, usually on monotherapy. Seizures in older adults tend to be more medically responsive.^53^ There is also a possibility that some seizures are unrecognized and that the medical responsiveness is being overestimated, as the seizure semiology in older adults can be very subtle. Indeed, one participant had subclinical seizures on ambulatory EEG during this study.

### Limitations

This study has several limitations: (i) The cohort mostly consisted of white individuals with a high level of education, affecting the generalizability of the findings. (ii) We did not have a visuospatial memory task as part of the cognitive battery; therefore, we are unable to determine the degree of visual memory impairment. (iii) The Harvard Aging Brain Study comparison group consists of relatively healthy community dwelling older adults, who did not have evidence of depression or cognitive impairment on enrolment. (iv) The study included individuals with isolated seizures who did not fulfil criteria for epilepsy. (v) The age cut-off of 55 is consistent with similar prior studies of late-onset epilepsy, although others have advocated a cut-off of 65.^54^

## Conclusions

Individuals with late-onset unexplained epilepsy show evidence of temporal dysfunction on EEG with left-sided predominance, with cognitive impairments mostly in the verbal memory domain. Neuroimaging shows lower amygdalae and white matter hyperintensity volumes compared with healthy controls. Future studies extending these findings to Alzheimer’s disease biomarkers and longitudinal follow-up will further inform predictors of cognitive decline.

## Supplementary Material

fcaf050_Supplementary_Data

## Data Availability

Anonymized data will be made available by request from any qualified investigator.

## References

[1] Hauser WA , Hesdorffer DC. Epilepsy: Frequency, causes, and consequences. Demos; 1990.

[2] Hauser WA , Annegers JF, Kurland LT. Prevalence of epilepsy in Rochester, Minnesota: 1940–1980. Epilepsia. 1991;32(4):429–445. 1868801 10.1111/j.1528-1157.1991.tb04675.x

[3] Arabi M , Dirani M, Hourani R, et al Frequency and stratification of epileptogenic lesions in elderly with new onset seizures. Front Neurol. 2018;9:995. 30559705 10.3389/fneur.2018.00995 PMC6284348

[4] Tanaka A , Akamatsu N, Shouzaki T, et al Clinical characteristics and treatment responses in new-onset epilepsy in the elderly. Seizure. 2013;22(9):772–775. 23849689 10.1016/j.seizure.2013.06.005

[5] Emsley HCA , Gibson LM, Allan SM, Parkes LM. Occult cerebrovascular disease and late-onset epilepsy: Could loss of neurovascular unit integrity be a viable model? Cardiovasc Psychiatry Neurol. 2011;2011:130406. 21461380 10.1155/2011/130406 PMC3063412

[6] Romoli M , Sen A, Parnetti L, Calabresi P, Costa C. Amyloid-β: A potential link between epilepsy and cognitive decline. Nat Rev Neurol. 2021;17(8):469–485. 34117482 10.1038/s41582-021-00505-9

[7] Cretin B , Sellal F, Philippi N, et al Epileptic prodromal Alzheimer’s disease, a retrospective study of 13 new cases: Expanding the spectrum of Alzheimer’s disease to an epileptic variant? J Alzheimers Dis. 2016;52(3):1125–1133. 27104892 10.3233/JAD-150096

[8] Chang CS , Liao CH, Lin CC, Lane HY, Sung FC, Kao CH. Patients with epilepsy are at an increased risk of subsequent stroke: A population-based cohort study. Seizure. 2014;23(5):377–381. 24630806 10.1016/j.seizure.2014.02.007

[9] Choi H , Thacker EL, Longstreth WT, Elkind MSV, Boehme AK. Cognitive decline in older adults with epilepsy: The cardiovascular health study. Epilepsia. 2021;62(1):85–97. 33227164 10.1111/epi.16748 PMC12432973

[10] Tai XY , Torzillo E, Lyall DM, Manohar S, Husain M, Sen A. Association of dementia risk with focal epilepsy and modifiable cardiovascular risk factors. JAMA Neurol. 2023;80(5):445–454. 36972059 10.1001/jamaneurol.2023.0339 PMC10043806

[11] Johnson EL , Krauss GL, Kucharska-Newton A, et al Dementia in late-onset epilepsy: The atherosclerosis risk in communities study. Neurology. 2020;95(24):E3248–E3256. 33097597 10.1212/WNL.0000000000011080 PMC7836657

[12] Piazzini A , Canevini MP, Turner K, Chifari R, Canger R. Elderly people and epilepsy: Cognitive function. Epilepsia. 2006;47(Suppl 5):82–84. 10.1111/j.1528-1167.2006.00884.x 17239113

[13] Liguori C , Costa C, Franchini F, et al Cognitive performances in patients affected by late-onset epilepsy with unknown etiology: A 12-month follow-up study. Epilepsy Behav. 2019;101:106592. 31726425 10.1016/j.yebeh.2019.106592

[14] Sarkis RA , Beers L, Farah E, et al The neurophysiology and seizure outcomes of late onset unexplained epilepsy. Clin Neurophysiol. 2020;131(11):2667–2672. 32957039 10.1016/j.clinph.2020.08.014 PMC7644268

[15] Nagino N , Kubota Y, Nakamoto H, et al Non-lesional late-onset epilepsy in the elderly Japanese patients: Presenting characteristics and seizure outcomes with regard to comorbid dementia. J Clin Neurosci. 2022;103:100–106. 35868225 10.1016/j.jocn.2022.05.003

[16] DiFrancesco JC , Isella V, Licciardo D, et al Temporal lobe dysfunction in late-onset epilepsy of unknown origin. Epilepsy Behav. 2021;117:107839. 33611099 10.1016/j.yebeh.2021.107839

[17] Thal DR , Rüb U, Orantes M, Braak H. Phases of A beta-deposition in the human brain and its relevance for the development of AD. Neurology. 2002;58(12):1791–1800. 12084879 10.1212/wnl.58.12.1791

[18] Braak H , Braak E. Neuropathological stageing of Alzheimer-related changes. Acta Neuropathol. 1991;82(4):239–259. 1759558 10.1007/BF00308809

[19] Vossel KA , Tartaglia MC, Nygaard HB, Zeman AZ, Miller BL. Epileptic activity in Alzheimer’s disease: Causes and clinical relevance. Lancet Neurol. 2017;16(4):311–322. 28327340 10.1016/S1474-4422(17)30044-3 PMC5973551

[20] Fisher RS , Acevedo C, Arzimanoglou A, et al ILAE official report: A practical clinical definition of epilepsy. Epilepsia. 2014;55(4):475–482. 24730690 10.1111/epi.12550

[21] Dagley A , LaPoint M, Huijbers W, et al Harvard aging brain study: Dataset and accessibility. Neuroimage. 2017;144(Pt B):255–258. 25843019 10.1016/j.neuroimage.2015.03.069 PMC4592689

[22] Wechsler D . WMS-R:Wechsler memory scale—Revised. Psychological Corporation; 1987.

[23] Grober E , Lipton RB, Hall C, Crystal H. Memory impairment on free and cued selective reminding predicts dementia. Neurology. 2000;54(4):827–832. 10690971 10.1212/wnl.54.4.827

[24] Wechsler D . WAIS-R Manual: Wechsler adult intelligence scale—Revised. Psychological Corporation; 1981.

[25] Wechsler D . WAIS-III Administration and scoring manual. Psychological Corporation; 1997.

[26] Papp KV , Rentz DM, Orlovsky I, Sperling RA, Mormino EC. Optimizing the preclinical Alzheimer’s cognitive composite with semantic processing: The PACC5. Alzheimers Dement (N Y). 2017;3(4):668–677. 29264389 10.1016/j.trci.2017.10.004 PMC5726754

[27] Donohue MC , Sperling RA, Salmon DP, et al The preclinical Alzheimer cognitive composite: Measuring amyloid-related decline. JAMA Neurol. 2014;71(8):961–970. 24886908 10.1001/jamaneurol.2014.803 PMC4439182

[28] Morris JC . The clinical dementia rating (CDR): Current version and scoring rules. Neurology. 1993;43(11):2412–2414. 10.1212/wnl.43.11.2412-a 8232972

[29] Yesavage JA , Brink TL, Rose TL, et al Development and validation of a geriatric depression screening scale: A preliminary report. J Psychiatr Res. 1982;17(1):37–49. 7183759 10.1016/0022-3956(82)90033-4

[30] Fischl B , Salat DH, Busa E, et al Whole brain segmentation: Automated labeling of neuroanatomical structures in the human brain. Neuron. 2002;33(3):341–355. 11832223 10.1016/s0896-6273(02)00569-x

[31] Schmidt P . Bayesian inference for structured additive regression models for large-scale problems with applications to medical imaging. PhD thesis. Ludwig-Maximilians-Universität München; 2017;Ch 6.1(November).

[32] Egger C , Opfer R, Wang C, et al MRI FLAIR lesion segmentation in multiple sclerosis: Does automated segmentation hold up with manual annotation? Neuroimage Clin. 2017;13:264–270. 28018853 10.1016/j.nicl.2016.11.020 PMC5175993

[33] Kwan P , Arzimanoglou A, Berg AT, et al Definition of drug resistant epilepsy: Consensus proposal by the ad hoc task force of the ILAE commission on therapeutic strategies. Epilepsia. 2009;51(6):1069–1077. 19889013 10.1111/j.1528-1167.2009.02397.x

[34] Johnson EL , Krauss GL, Walker KA, et al Late-onset epilepsy and 25-year cognitive change: The atherosclerosis risk in communities (ARIC) study. Epilepsia. 2020;61(8):1764–1773. 32710450 10.1111/epi.16616 PMC7718433

[35] Costa C , Romoli M, Liguori C, et al Alzheimer’s disease and late-onset epilepsy of unknown origin: Two faces of beta amyloid pathology. Neurobiol Aging. 2019;73:61–67. 30317034 10.1016/j.neurobiolaging.2018.09.006

[36] Reyes A , Schneider ALC, Kucharska-Newton AM, Gottesman RF, Johnson EL, McDonald CR. Cognitive phenotypes in late-onset epilepsy: Results from the atherosclerosis risk in communities study. Front Neurol. 2023;14:1230368. 37745655 10.3389/fneur.2023.1230368 PMC10513940

[37] Sarkis RA , McGinnis S, Rushia SN, Park S, Ansari EE, Willment KC. Growing older with drug-resistant epilepsy: Cognitive and psychosocial outcomes. J Neurol. 2018;265(5):1059–1064. 29478222 10.1007/s00415-018-8805-z

[38] Bai W , Chen P, Cai H, et al Worldwide prevalence of mild cognitive impairment among community dwellers aged 50 years and older: A meta-analysis and systematic review of epidemiology studies. Age Ageing. 2022;51(8):afac173. 35977150 10.1093/ageing/afac173

[39] Gugger JJ , Walter AE, Diaz-Arrastia R, et al Association between structural brain MRI abnormalities and epilepsy in older adults. Ann Clin Transl Neurol. 2024;11(2):342–354. 38155477 10.1002/acn3.51955 PMC10863905

[40] Kaestner E , Reyes A, Chen A, et al Atrophy and cognitive profiles in older adults with temporal lobe epilepsy are similar to mild cognitive impairment. Brain. 2021;144(1):236–250. 33279986 10.1093/brain/awaa397 PMC7880670

[41] Nelson PT , Abner EL, Patel E, et al The amygdala as a locus of pathologic misfolding in neurodegenerative diseases. J Neuropathol Exp Neurol. 2018;77(1):2–20. 29186501 10.1093/jnen/nlx099 PMC5901077

[42] Foldvary-Schaefer N , Unnwongse K. Localizing and lateralizing features of auras and seizures. Epilepsy Behav. 2011;20(2):160–166. 20926350 10.1016/j.yebeh.2010.08.034

[43] Roddy D , Kelly JR, Farrell C, et al Amygdala substructure volumes in major depressive disorder. Neuroimage Clin. 2021;31:102781. 34384996 10.1016/j.nicl.2021.102781 PMC8361319

[44] Schmaal L , Veltman DJ, Van Erp TGM, et al Subcortical brain alterations in major depressive disorder: Findings from the ENIGMA major depressive disorder working group. Mol Psychiatry. 2016;21(6):806–812. 26122586 10.1038/mp.2015.69 PMC4879183

[45] Johnson EL , Sullivan KJ, Schneider ALC, et al Association of plasma Aβ42/Aβ40 ratio and late-onset epilepsy: Results from the atherosclerosis risk in communities study. Neurology. 2023;101(13):E1319–E1327. 37541842 10.1212/WNL.0000000000207635 PMC10558158

[46] Kjeldsen PL , Parbo P, Hansen KV, et al Asymmetric amyloid deposition in preclinical Alzheimer’s disease: A PET study. Aging Brain. 2022;2:100048. 36908895 10.1016/j.nbas.2022.100048 PMC9997142

[47] Vogel JW , Young AL, Oxtoby NP, et al Four distinct trajectories of tau deposition identified in Alzheimer’s disease. Nat Med. 2021;27(5):871–881. 33927414 10.1038/s41591-021-01309-6 PMC8686688

[48] Sarkis RA , Willment KC, Pennell PB, Marshall G. Late-onset unexplained epilepsy: What are we missing? Epilepsy Behav. 2019;99:106478. 31481308 10.1016/j.yebeh.2019.106478

[49] Johnson EL , Krauss GL, Lee AK, et al Association between white matter hyperintensities, cortical volumes, and late-onset epilepsy. Neurology. 2019;92(9):E988–E995. 30804067 10.1212/WNL.0000000000007010 PMC6404466

[50] Morrison C , Dadar M, Manera AL, Collins DL. Racial differences in white matter hyperintensity burden in older adults. Neurobiol Aging. 2023;122:112–119. 36543016 10.1016/j.neurobiolaging.2022.11.012

[51] Yao Z , Hu B, Liang C, Zhao L, Jackson M; Alzheimer’s disease neuroimaging initiative. A longitudinal study of atrophy in amnestic mild cognitive impairment and normal aging revealed by cortical thickness. PLoS One. 2012;7(11):e48973. 23133666 10.1371/journal.pone.0048973 PMC3487850

[52] Vossel KA , Ranasinghe KG, Beagle AJ, et al Incidence and impact of subclinical epileptiform activity in Alzheimer’s disease. Ann Neurol. 2016;80:858–870. 27696483 10.1002/ana.24794 PMC5177487

[53] Brodie MJ , Stephen LJ. Outcomes in elderly patients with newly diagnosed and treated epilepsy. Int Rev Neurobiol. 2007;81:253–263. 17433929 10.1016/S0074-7742(06)81016-0

[54] Josephson CB , Engbers JDT, Sajobi TT, et al Towards a clinically informed, data-driven definition of elderly onset epilepsy. Epilepsia. 2016;57(2):298–305. 26648047 10.1111/epi.13266

