# Supplementary material for: Late-onset unexplained seizures are associated with cognitive impairment and lower amygdala volumes
Source: Brain Commun. 2025 Jan 31;7(1):fcaf050. doi: 10.1093/braincomms/fcaf050 (PMC11815171; doi:10.1093/braincomms/fcaf050)
Supplement: fcaf050_Supplementary_Data [file fcaf050_supplementary_data.docx]

Supplementary Figure 1. Study flow diagram

EMU: epilepsy monitoring unit, LOUE: late onset unexplained epilepsy

Model 1. Predictors of **% normalized left hippocampal volume** while controlling for age, sex, race.

| **Term** | **Estimate** | **Prob>\|t\|** | **Lower 95%** | **Upper 95%** |
| --- | --- | --- | --- | --- |
| Intercept | 0.3667029 | <.0001* | 0.3373583 | 0.3960476 |
| Cohort[LOUE] | 0.0007529 | 0.7043 | -0.003144 | 0.0046494 |
| Age | -0.001608 | <.0001* | -0.00198 | -0.001236 |
| Sex[F] | 0.0071929 | <.0001* | 0.0043413 | 0.0100444 |
| Race[AS] | 0.0097614 | 0.2897 | -0.008339 | 0.0278617 |
| Race[B] | -0.001825 | 0.7748 | -0.014356 | 0.0107065 |
| Race[O] | -0.003599 | 0.8156 | -0.033914 | 0.0267153 |

As: Asian, B: Black, F: female, LOUE: late onset unexplained epilepsy, O: other

R^2^=0.25, *p<0.05

Model 2. Predictors of **% normalized right hippocampal volume** while controlling for age, sex, race.

| **Term** | **Estimate** | **Prob>\|t\|** | **Lower 95%** | **Upper 95%** |
| --- | --- | --- | --- | --- |
| Intercept | 0.3645539 | <.0001* | 0.3349387 | 0.394169 |
| Cohort[LOUE] | -0.003131 | 0.1184 | -0.007063 | 0.0008018 |
| Age | -0.001538 | <.0001* | -0.001914 | -0.001163 |
| Sex[F] | 0.0085 | <.0001* | 0.0056222 | 0.0113778 |
| Race[AS] | 0.0145065 | 0.1193 | -0.003761 | 0.0327736 |
| Race[B] | -0.000178 | 0.9779 | -0.012825 | 0.0124683 |
| Race[O] | -0.009429 | 0.5449 | -0.040024 | 0.0211648 |

As: Asian, B: Black, F: female, LOUE: late onset unexplained epilepsy, O: other

R^2^=0.23*p<0.05

Model 3. Predictors of **% normalized left amygdala** **volume** while controlling for age, sex, race.

| **Term** | **Estimate** | **Prob>\|t\|** | **Lower 95%** | **Upper 95%** |
| --- | --- | --- | --- | --- |
| Intercept | 0.1669751 | <.0001* | 0.1522448 | 0.1817054 |
| Cohort[LOUE] | -0.003152 | 0.0016* | -0.005108 | -0.001196 |
| Age | -0.000991 | <.0001* | -0.001178 | -0.000804 |
| Sex[F] | -0.000541 | 0.4582 | -0.001972 | 0.0008907 |
| Race[AS] | -0.000306 | 0.9472 | -0.009392 | 0.0087798 |
| Race[B] | 0.0001107 | 0.9724 | -0.00618 | 0.0064011 |
| Race[O] | 0.0016345 | 0.8329 | -0.013583 | 0.0168517 |

As: Asian, B: Black, F: female, LOUE: late onset unexplained epilepsy, O: other

R^2^=0.22, *p<0.05

Model 4. Predictors of **% normalized right amygdala** volume while controlling for age, sex, race.

| **Term** | **Estimate** | **Prob>\|t\|** | **Lower 95%** | **Upper 95%** |
| --- | --- | --- | --- | --- |
| Intercept | 0.154693 | <.0001* | 0.1399023 | 0.1694837 |
| Cohort[LOUE] | -0.00259 | 0.0099* | -0.004554 | -0.000626 |
| Age | -0.000689 | <.0001* | -0.000876 | -0.000501 |
| Sex[F] | -0.000242 | 0.7409 | -0.001679 | 0.0011954 |
| Race[AS] | 0.0011812 | 0.7992 | -0.007942 | 0.0103044 |
| Race[B] | 0.0034986 | 0.2769 | -0.002818 | 0.0098148 |
| Race[O] | -0.003759 | 0.6289 | -0.019039 | 0.0115203 |

As: Asian, B: Black, F: female, LOUE: late onset unexplained epilepsy, O: other

R^2^=0.14, *p<0.05

Model 5. Predictors of **log transformed white matter hyperintensity volume** while controlling for age, sex, race.

| **Term** | **Estimate** | **Prob>\|t\|** | **Lower 95%** | **Upper 95%** |
| --- | --- | --- | --- | --- |
| Intercept | -1.552826 | 0.0374* | -3.014473 | -0.091179 |
| Cohort[LOUE] | -0.21319 | 0.0301* | -0.405718 | -0.020661 |
| Age | 0.1173739 | <.0001* | 0.0988255 | 0.1359224 |
| Sex[F] | 0.0453945 | 0.5274 | -0.095674 | 0.1864628 |
| Race[AS] | -0.049961 | 0.9120 | -0.937889 | 0.8379671 |
| Race[B] | 0.6716797 | 0.0328* | 0.0551359 | 1.2882235 |
| Race[O] | -0.864678 | 0.2536 | -2.351549 | 0.6221922 |

As: Asian, B: Black, F: female, LOUE: late onset unexplained epilepsy, O: other

R^2^=0.32, *p<0.05

**Sensitivity Analyses.**

Model 6. Predictors of **% normalized left hippocampal volume** while controlling for age, sex, race, and vascular risk factors.

| **Term** | **Estimate** | **Prob>\|t\|** | **Lower 95%** | **Upper 95%** |
| --- | --- | --- | --- | --- |
| Intercept | 0.3673204 | <.0001* | 0.3289709 | 0.4056698 |
| Cohort[LOUE] | 0.0016182 | 0.4235 | -0.002353 | 0.0055894 |
| Age | -0.001539 | <.0001* | -0.001941 | -0.001137 |
| Sex[F] | 0.0079707 | <.0001* | 0.005015 | 0.0109263 |
| Race [AS] | 0.0097829 | 0.2892 | -0.008341 | 0.0279063 |
| Race [B] | -4.945e-5 | 0.9939 | -0.012862 | 0.0127634 |
| Race [O] | -0.002283 | 0.8820 | -0.032499 | 0.0279326 |
| DM[N] | 8.9071e-5 | 0.9729 | -0.005055 | 0.0052328 |
| HTN[N] | 0.003079 | 0.0532 | -4.316e-5 | 0.0062012 |
| Smoking[N] | -0.002722 | 0.4648 | -0.010036 | 0.0045927 |
| BMI | -1.626e-5 | 0.9615 | -0.000677 | 0.0006447 |

R^2^=0.24, *p<0.05.

As: Asian, B: Black, BMI: Body mass index, DM: Diabetes Mellitus, F: female, HTN: Hypertension, LOUE: Late onset unexplained epilepsy, N: No, O: other.

Model 7. Predictors of **% normalized right hippocampal** volume while controlling for age, sex, race, and vascular risk factors.

| **Term** | **Estimate** | **Prob>\|t\|** | **Lower 95%** | **Upper 95%** |
| --- | --- | --- | --- | --- |
| Intercept | 0.3568174 | <.0001* | 0.318259 | 0.3953758 |
| Cohort[LOUE] | -0.002881 | 0.1568 | -0.006874 | 0.0011117 |
| Age | -0.001483 | <.0001* | -0.001887 | -0.001078 |
| Sex[F] | 0.0094288 | <.0001* | 0.006457 | 0.0124006 |
| Race [AS] | 0.0150513 | 0.1052 | -0.003171 | 0.0332735 |
| Race [B] | 0.0007467 | 0.9093 | -0.012136 | 0.0136294 |
| Race [O] | -0.009435 | 0.5418 | -0.039815 | 0.0209457 |
| DM[N] | 0.0007744 | 0.7686 | -0.004397 | 0.0059462 |
| HTN[N] | 0.0020708 | 0.1954 | -0.001068 | 0.00521 |
| Smoking[N] | -0.004973 | 0.1845 | -0.012327 | 0.0023813 |
| BMI | 0.0003353 | 0.3218 | -0.000329 | 0.0009999 |

R^2^=0.26, *p<0.05.

As: Asian, B: Black, BMI: Body mass index, DM: Diabetes Mellitus, F: female, HTN: Hypertension, LOUE: Late onset unexplained epilepsy, N: No, O: other.

Model 8. Predictors of **% normalized right amygdala volume** while controlling for age, sex, race, and vascular risk factors.

| **Term** | **Estimate** | **Prob>\|t\|** | **Lower 95%** | **Upper 95%** |
| --- | --- | --- | --- | --- |
| Intercept | 0.1416038 | <.0001* | 0.1224452 | 0.1607624 |
| Cohort[LOUE] | -0.002752 | 0.0067* | -0.004736 | -0.000768 |
| Age | -0.000675 | <.0001* | -0.000875 | -0.000474 |
| Sex[F] | 0.0001522 | 0.8395 | -0.001324 | 0.0016288 |
| Race [AS] | 0.002447 | 0.5954 | -0.006607 | 0.0115012 |
| Race [B] | 0.0035431 | 0.2771 | -0.002858 | 0.0099441 |
| Race [O] | -0.004877 | 0.5256 | -0.019972 | 0.0102181 |
| DM[N] | 0.000247 | 0.8502 | -0.002323 | 0.0028167 |
| HTN[N] | 0.0009524 | 0.2307 | -0.000607 | 0.0025121 |
| Smoking[N] | 0.0004997 | 0.7882 | -0.003154 | 0.0041537 |
| BMI | 0.0004365 | 0.0097* | 0.0001063 | 0.0007667 |

R^2^=0.18, *p<0.05.

As: Asian, B: Black, BMI: Body mass index, DM: Diabetes Mellitus, F: female, HTN: Hypertension, LOUE: Late onset unexplained epilepsy, N: No, O: other.

Model 9. Predictors of **% normalized left amygdala** **volume** while controlling for age, sex, race, and vascular risk factors.

| **Term** | **Estimate** | **Prob>\|t\|** | **Lower 95%** | **Upper 95%** |
| --- | --- | --- | --- | --- |
| Intercept | 0.1555398 | <.0001* | 0.1364123 | 0.1746673 |
| Cohort[LOUE] | -0.002918 | 0.0040* | -0.004899 | -0.000937 |
| Age | -0.000962 | <.0001* | -0.001163 | -0.000762 |
| Sex[F] | -0.000122 | 0.8703 | -0.001597 | 0.0013517 |
| Race [AS] | 0.00107 | 0.8161 | -0.007969 | 0.0101094 |
| Race [B] | 0.000414 | 0.8987 | -0.005977 | 0.0068047 |
| Race [O] | 0.0009837 | 0.8979 | -0.014087 | 0.0160544 |
| DM[N] | 0.0002391 | 0.8547 | -0.002326 | 0.0028047 |
| HTN[N] | 0.0014404 | 0.0698 | -0.000117 | 0.0029976 |
| Smoking[N] | 0.0031797 | 0.0874 | -0.000468 | 0.0068278 |
| BMI | 0.0002721 | 0.1055 | -5.758e-5 | 0.0006017 |

R^2^=0.25, *p<0.05.

As: Asian, B: Black, BMI: Body mass index, DM: Diabetes Mellitus, F: female, HTN: Hypertension, LOUE: Late onset unexplained epilepsy, N: No, O: other.

Model 10. Predictors of **log transformed white matter hyperintensity volume** while controlling for age, sex, race, and vascular risk factors.

| **Term** | **Estimate** | **Prob>\|t\|** | **Lower 95%** | **Upper 95%** |
| --- | --- | --- | --- | --- |
| Intercept | -1.4208 | 0.1346 | -3.284182 | 0.4425825 |
| Cohort[LOUE] | -0.255223 | 0.0097* | -0.448211 | -0.062235 |
| Age | 0.1101794 | <.0001* | 0.0905348 | 0.129824 |
| Sex[F] | 0.0536892 | 0.4627 | -0.089905 | 0.1972837 |
| Race [AS] | -0.059167 | 0.8944 | -0.934729 | 0.8163953 |
| Race [B] | 0.5550697 | 0.0797 | -0.066014 | 1.1761532 |
| Race [O] | -0.975146 | 0.1897 | -2.434492 | 0.4842006 |
| DM[N] | -0.043437 | 0.7375 | -0.298047 | 0.2111736 |
| HTN[N] | -0.24648 | 0.0016* | -0.398644 | -0.094315 |
| Smoking[N] | -0.042309 | 0.8141 | -0.395935 | 0.3113172 |
| BMI | 0.0101246 | 0.5361 | -0.022021 | 0.0422702 |

R^2^=0.32, *p<0.05.

As: Asian, B: Black, BMI: Body mass index, DM: Diabetes Mellitus, F: female, HTN: Hypertension, LOUE: Late onset unexplained epilepsy, N: No, O: other.

Model 11. Predictors of **% normalized left hippocampal volume** while controlling for age, sex, race, global cognition, and depression.

| **Term** | **Estimate** | **Prob>\|t\|** | **Lower 95%** | **Upper 95%** |
| --- | --- | --- | --- | --- |
| Intercept | 0.3305483 | <.0001* | 0.256115 | 0.4049815 |
| Cohort[LOUE] | -7.621e-5 | 0.9715 | -0.004271 | 0.0041189 |
| Age | -0.001536 | <.0001* | -0.001918 | -0.001154 |
| Sex[F] | 0.0078052 | <.0001* | 0.0049506 | 0.0106598 |
| Race [AS] | 0.0103898 | 0.2555 | -0.007547 | 0.0283266 |
| Race [B] | -0.002048 | 0.7478 | -0.014561 | 0.0104653 |
| Race [O] | -0.003148 | 0.8373 | -0.033263 | 0.0269665 |
| MMSE | 0.0010794 | 0.3490 | -0.001184 | 0.0033427 |
| GDS | -0.000131 | 0.7529 | -0.000948 | 0.0006861 |

R^2^=0.31, *p<0.05.

As: Asian, B: Black, F: female, GDS: geriatric depression scale, HTN: Hypertension, LOUE: Late onset unexplained epilepsy, MMSE: Mini mental status exam, O: other.

Model 12. Predictors of **% normalized right hippocampal** volume while controlling for age, sex, race, global cognition, and depression.

| **Term** | **Estimate** | **Prob>\|t\|** | **Lower 95%** | **Upper 95%** |
| --- | --- | --- | --- | --- |
| Intercept | 0.3079979 | <.0001* | 0.2319783 | 0.3840175 |
| Cohort[LOUE] | -0.003614 | 0.0981 | -0.007898 | 0.0006709 |
| Age | -0.001479 | <.0001* | -0.001869 | -0.001089 |
| Sex[F] | 0.0087689 | <.0001* | 0.0058535 | 0.0116844 |
| Race [AS] | 0.0148511 | 0.1118 | -0.003468 | 0.0331701 |
| Race [B] | -0.000615 | 0.9246 | -0.013395 | 0.0121648 |
| Race [O] | -0.007794 | 0.6186 | -0.03855 | 0.0229623 |
| MMSE | 0.0018127 | 0.1240 | -0.000499 | 0.0041242 |
| GDS | 0.0001996 | 0.6384 | -0.000635 | 0.001034 |

R^2^=0.23, *p<0.05.

As: Asian, B: Black, F: female, GDS: geriatric depression scale, HTN: Hypertension, LOUE: Late onset unexplained epilepsy, MMSE: Mini mental status exam, O: other.

Model 13. Predictors of **% normalized right amygdala volume** while controlling for age, sex, race, global cognition, and depression.

| **Term** | **Estimate** | **Prob>\|t\|** | **Lower 95%** | **Upper 95%** |
| --- | --- | --- | --- | --- |
| Intercept | 0.1351093 | <.0001* | 0.0971247 | 0.173094 |
| Cohort[LOUE] | -0.00277 | 0.0113* | -0.004911 | -0.000629 |
| Age | -0.000674 | <.0001* | -0.000869 | -0.000479 |
| Sex[F] | -0.000162 | 0.8271 | -0.001619 | 0.0012948 |
| Race [AS] | 0.0012891 | 0.7820 | -0.007864 | 0.0104425 |
| Race [B] | 0.003499 | 0.2820 | -0.002887 | 0.0098848 |
| Race [O] | -0.003283 | 0.6747 | -0.018651 | 0.0120851 |
| MMSE | 0.0006468 | 0.2716 | -0.000508 | 0.0018018 |
| GDS | 0.000026 | 0.9025 | -0.000391 | 0.0004429 |

R^2^=0.14, *p<0.05.

As: Asian, B: Black, F: female, GDS: geriatric depression scale, HTN: Hypertension, LOUE: Late onset unexplained epilepsy, MMSE: Mini mental status exam, O: other.

Model 14. Predictors of **% normalized left amygdala** **volume** while controlling for age, sex, race, global cognition, and depression.

| **Term** | **Estimate** | **Prob>\|t\|** | **Lower 95%** | **Upper 95%** |
| --- | --- | --- | --- | --- |
| Intercept | 0.1453986 | <.0001* | 0.1088029 | 0.1819943 |
| Cohort[LOUE] | -0.003785 | 0.0003* | -0.005848 | -0.001723 |
| Age | -0.000941 | <.0001* | -0.001129 | -0.000754 |
| Sex[F] | -0.000248 | 0.7289 | -0.001651 | 0.0011558 |
| Race [AS] | 0.0001421 | 0.9747 | -0.008677 | 0.0089608 |
| Race [B] | -0.000395 | 0.8997 | -0.006547 | 0.0057576 |
| Race [O] | 0.002175 | 0.7729 | -0.012631 | 0.0169811 |
| MMSE | 0.0006085 | 0.2830 | -0.000504 | 0.0017213 |
| GDS | 0.0000208 | 0.9190 | -0.000381 | 0.0004225 |

R^2^=0.23, *p<0.05.

As: Asian, B: Black, F: female, GDS: geriatric depression scale, HTN: Hypertension, LOUE: Late onset unexplained epilepsy, MMSE: Mini mental status exam, O: other.

Model 15. Predictors of **log transformed white matter hyperintensity volume** while controlling for age, sex, race, global cognition, and depression.

| **Term** | **Estimate** | **Prob>\|t\|** | **Lower 95%** | **Upper 95%** |
| --- | --- | --- | --- | --- |
| Intercept | 0.7207085 | 0.7044 | -3.011056 | 4.452473 |
| Cohort[LOUE] | -0.246133 | 0.0215* | -0.455749 | -0.036517 |
| Age | 0.1163609 | <.0001* | 0.0970313 | 0.1356904 |
| Sex[F] | 0.0477817 | 0.5122 | -0.095402 | 0.1909654 |
| Race [AS] | -0.05314 | 0.9070 | -0.946532 | 0.8402509 |
| Race [B] | 0.6350398 | 0.0466* | 0.0096675 | 1.260412 |
| Race [O] | -0.897756 | 0.2399 | -2.397471 | 0.6019587 |
| MMSE | -0.080424 | 0.1633 | -0.193641 | 0.0327929 |
| GDS | 0.0129702 | 0.5372 | -0.028317 | 0.0542579 |

R^2^=0.30, *p<0.05.

As: Asian, B: Black, F: female, GDS: geriatric depression scale, HTN: Hypertension, LOUE: Late onset unexplained epilepsy, MMSE: Mini mental status exam, O: other.

Model 16. Predictors of **% normalized left hippocampal volume** while controlling for age, sex, race, clinical dementia rating.

| **Term** | **Estimate** | **Prob>\|t\|** | **Lower 95%** | **Upper 95%** |
| --- | --- | --- | --- | --- |
| Intercept | 0.3657388 | <.0001* | 0.3362794 | 0.3951982 |
| Cohort[LOUE] | 0.0016835 | 0.4548 | -0.002739 | 0.0061064 |
| Age at testing | -0.001578 | <.0001* | -0.001952 | -0.001205 |
| Gender[F] | 0.0073101 | <.0001* | 0.0044564 | 0.0101639 |
| Race 2[AS] | 0.0102123 | 0.2679 | -0.007883 | 0.0283075 |
| Race 2[B] | -0.001752 | 0.7834 | -0.014274 | 0.0107696 |
| Race 2[O] | -0.003904 | 0.8001 | -0.034195 | 0.0263874 |
| CDR [0.5-0] | -0.00729 | 0.2581 | -0.019945 | 0.0053653 |

R^2^=0.21, *p<0.05.

As: Asian, B: Black, CDR: Clinical dementia rating, F: female, HTN: Hypertension, LOUE: Late onset unexplained epilepsy, O: other.

Model 17. Predictors of **% normalized right hippocampal** volume while controlling for age, sex, race, clinical dementia rating.

| **Term** | **Estimate** | **Prob>\|t\|** | **Lower 95%** | **Upper 95%** |
| --- | --- | --- | --- | --- |
| Intercept | 0.3646742 | <.0001* | 0.3348603 | 0.3944881 |
| Cohort[LOUE] | -0.003682 | 0.1067 | -0.008158 | 0.0007945 |
| Age | -0.00155 | <.0001* | -0.001928 | -0.001172 |
| Sex[F] | 0.008462 | <.0001* | 0.0055739 | 0.0113501 |
| Race [AS] | 0.0142915 | 0.1258 | -0.004021 | 0.0326045 |
| Race [B] | -0.000227 | 0.9719 | -0.0129 | 0.0124454 |
| Race [O] | -0.009276 | 0.5523 | -0.039932 | 0.0213794 |
| CDR [0.5-0] | 0.0038405 | 0.5559 | -0.008967 | 0.016648 |

R^2^=0.22, *p<0.05.

As: Asian, B: Black, CDR: Clinical dementia rating, F: female, HTN: Hypertension, LOUE: Late onset unexplained epilepsy, O: other.

Model 18. Predictors of **% normalized right amygdala volume** while controlling for age, sex, race, clinical dementia rating.

| **Term** | **Estimate** | **Prob>\|t\|** | **Lower 95%** | **Upper 95%** |
| --- | --- | --- | --- | --- |
| Intercept | 0.1547173 | <.0001* | 0.139835 | 0.1695996 |
| Cohort[LOUE] | -0.002163 | 0.0577 | -0.004398 | 7.094e-5 |
| Age at testing | -0.000682 | <.0001* | -0.00087 | -0.000493 |
| Gender[F] | -0.000221 | 0.7636 | -0.001662 | 0.001221 |
| Race 2[AS] | 0.0013341 | 0.7743 | -0.007807 | 0.0104754 |
| Race 2[B] | 0.0035378 | 0.2722 | -0.002788 | 0.0098635 |
| Race 2[O] | -0.003871 | 0.6193 | -0.019173 | 0.0114319 |
| CDR [0.5-0] | -0.002848 | 0.3817 | -0.009241 | 0.0035454 |

R^2^=0.14, *p<0.05.

As: Asian, B: Black, CDR: Clinical dementia rating, F: female, HTN: Hypertension, LOUE: Late onset unexplained epilepsy, O: other.

Model 19. Predictors of **% normalized left amygdala** **volume** while controlling for age, sex, race, clinical dementia rating.

| **Term** | **Estimate** | **Prob>\|t\|** | **Lower 95%** | **Upper 95%** |
| --- | --- | --- | --- | --- |
| Intercept | 0.1663822 | <.0001* | 0.1515775 | 0.1811869 |
| Cohort[LOUE] | -0.002924 | 0.0100* | -0.005147 | -0.000702 |
| Age | -0.000978 | <.0001* | -0.001166 | -0.000791 |
| Sex[F] | -0.000487 | 0.5048 | -0.001921 | 0.0009471 |
| Race [AS] | -0.000155 | 0.9734 | -0.009248 | 0.0089392 |
| Race [B] | 0.0001239 | 0.9691 | -0.006169 | 0.0064167 |
| Race [O] | 0.0015383 | 0.8426 | -0.013684 | 0.0167611 |
| CDR [0.5-0] | -0.002163 | 0.5042 | -0.008523 | 0.0041972 |

R^2^=0.22, *p<0.05.

As: Asian, B: Black, CDR: Clinical dementia rating, F: female, HTN: Hypertension, LOUE: Late onset unexplained epilepsy, O: other.

Model 20. Predictors of **log transformed white matter hyperintensity volume** while controlling for age, sex, race, clinical dementia rating.

| **Term** | **Estimate** | **Prob>\|t\|** | **Lower 95%** | **Upper 95%** |
| --- | --- | --- | --- | --- |
| Intercept | -1.56976 | 0.0361* | -3.037275 | -0.102246 |
| Cohort[LOUE] | -0.292953 | 0.0088* | -0.511792 | -0.074114 |
| Age | 0.1162395 | <.0001* | 0.0976371 | 0.1348419 |
| Sex[F] | 0.0389387 | 0.5879 | -0.102232 | 0.1801094 |
| Race [AS] | -0.081188 | 0.8573 | -0.9685 | 0.8061246 |
| Race [B] | 0.6646171 | 0.0345* | 0.0488826 | 1.2803516 |
| Race [O] | -0.843843 | 0.2646 | -2.328733 | 0.6410461 |
| CDR [0.5-0] | 0.5447036 | 0.0973 | -0.099527 | 1.1889338 |

R^2^=0.30, *p<0.05.

As: Asian, B: Black, CDR: Clinical dementia rating, F: female, HTN: Hypertension, LOUE: Late onset unexplained epilepsy, O: other.
